# Rhinitis, Ocular, Throat and Dermal Symptoms, Headache and Tiredness among Students in Schools from Johor Bahru, Malaysia: Associations with Fungal DNA and Mycotoxins in Classroom Dust

**DOI:** 10.1371/journal.pone.0147996

**Published:** 2016-02-01

**Authors:** Dan Norbäck, Jamal Hisham Hashim, Gui-Hong Cai, Zailina Hashim, Faridah Ali, Erica Bloom, Lennart Larsson

**Affiliations:** 1 Department of Medical Science, Occupational and Environmental Medicine, Uppsala University and University Hospital, Uppsala, Sweden; 2 United Nations University-International Institute for Global Health, Kuala Lumpur, Malaysia; 3 Department of Community Health, National University of Malaysia, Kuala Lumpur, Malaysia; 4 Department of Environmental and Occupational Health, Faculty of Medicine, and Health Science, Universiti Putra Malaysia, Serdang, Selangor, Malaysia; 5 Primary Care Unit, Johor State Health Department, Johor Bahru, Malaysia; 6 IVL Swedish Environmental Research Institute Ltd., Stockholm, Sweden; 7 Department of Laboratory Medicine, Division of Medical Microbiology, University of Lund, Lund, Sweden; Telethon Institute for Child Health Research, AUSTRALIA

## Abstract

There are few studies on rhinitis and sick building syndrome (SBS) among students in tropical countries. We studied associations between levels of five fungal DNA sequences, two mycotoxins (sterigmatocystin and verrucarol) and cat allergen (Fel d 1) levels in schools and rhinitis and other weekly SBS symptoms in the students. Fungal DNA was measured by quantitative PCR and cat allergen by ELISA. Pupils (N = 462) from eight randomly selected schools in Johor Bahru, Malaysia participated (96%). Dust samples were collected by cotton swabs and Petri dishes exposed for one week. None of the schools had a mechanical ventilation system, but all classrooms had openable windows that were kept open during lectures and indoor CO_2_ levels were low (mean 492 ppm; range 380–690 ppm). Weekly nasal symptoms (rhinitis) (18.8%), ocular (11.6%), throat (11.1%), dermal symptoms, headache (20.6%) and tiredness (22.1%) were common. Total fungal DNA in swab samples was associated with rhinitis (p = 0.02), ocular symptoms (p = 0.009) and tiredness (p = 0.001). There were positive associations between *Aspergillus versicolor* DNA in Petri dish samples, ocular symptoms (p = 0.02) and tiredness (p = 0.001). The level of the mycotoxin verrucarol (produced by *Stachybotrys chartarum*) in swab samples was positively associated with tiredness (p = 0.04). *Streptomyces* DNA in swab samples (p = 0.03) and Petri dish samples (p = 0.03) were negatively associated with tiredness. In conclusion, total fungal contamination, measured as total fungal DNA) in the classrooms, *Aspergillus versicolor* and verrucarol can be risk factors for rhinitis and SBS symptoms among students in the tropical country Malaysia.

## Introduction

There is an increasing concern about health problems caused by the indoor environment. The term “sick building syndrome” (SBS) has been used to describe symptoms (ocular, nasal, throat and dermal symptoms, headache and tiredness) that can be influenced by the indoor environment [1]. Epidemiological studies have indicated that microbial compounds, volatile organic compounds (VOC)) and low ventilation flow can be associated with SBS symptoms [2–5]. Moreover, female gender, allergy (atopy) and asthma can been associated with SBS symptoms [5–7]. In addition, building dampness and indoor mould growth have been associated with SBS symptoms [8–9].

The school environment is an important indoor environment for children. Schools can be contaminated by a mixture of air pollutants including mould, bacteria, allergens, particles, VOC and formaldehyde [10–11]. One review concluded that schools might be an important source of allergen exposure, including cat and dog allergens [12]. Most school environment studies are from industrialized countries in temperate climate zones [10–12].

In tropical areas, indoor levels of mould can be high due to the warm and humid climate. There are different methods to measure indoor mould. One recent systematic review concluded that there is a need for more epidemiological studies measuring mould derived components in dust, including those measured by molecular methods [13]. Quantitative Polymerase Chain Reaction (qPCR or sometimes called real time PCR) can been used to detect indoor mould, irrespectively of viability [14,15]. Mycotoxins are secondary metabolites from mould. However, most toxicological data for mycotoxins are from *in vitro* cell tests or bioassays and human or animal toxicity data is limited [16]. Low levels of mycotoxins can be detected in dust from indoor environments by modern mass spectrometric methods [17]. One recent school environment study from three European countries (HITEA) measured a large number of mycotoxins, described as secondary fungal metabolites, in school dust [18]. Sterigmatocystin is a carcinogenic mycotoxin produced mainly by *Aspergillus versicolor* (*A*. *versicolor*). Other examples of mycotoxins are verrucarol and trichodermol, hydrolysis products of macrocyclic trichothecenes (including satratoxins) and trichodermin, mainly produced by *Stachybotrys chartarum* [19, 20]. Aflatoxins are mainly produced by *Aspergillus spp*., including *A*. *versicolor* and *A*. *flavus* [21]. There are few epidemiological studies on health effects of mycotoxins in indoor environments.

SBS studies among school children in Asia are rare and even less studies are available on associations between indoor mould exposure and SBS symptoms in tropical countries. Building dampness was associated with eye irritation, cough and tiredness among office workers in Taipei, Taiwan [22]. One office study from Mauritius reported that the concentration of airborne viable mould in offices were associated with SBS symptoms [23]. Some studies on SBS symptoms among school children are available from Japan, Korea and China. One study from Japan investigated associations between the school environment and home environment and SBS among junior high school students. High relative air humidity in the classrooms was associated with SBS [24]. One study from South Korea investigated headache and tiredness in primary school children but found no associations between these symptoms and CO_2_ concentration in the classrooms [25]. Two studies from China investigated associations between SBS among students and the school environment [26–27]. One study found negative (protective) associations between bacterial markers in vacuumed dust (endotoxin and muramic acid) and new onset of SBS symptoms. New onset of school-related symptoms was positively associated with total fungal DNA [26]. The other study found positive associations between environmental pollutants, including PM10, SO_2_ and NO_2_, and new onset of SBS symptoms [27]. We found no previous study on associations between indoor levels of allergens, fungal DNA or mycotoxins in schools in tropical areas and rhinitis or SBS symptoms.

Most studies on SBS have grouped the symptoms in three groups; mucosal symptoms, dermal symptoms and general symptoms [7, 26–27] and have studied weekly symptoms. Rhinitis is included in the group of mucosal symptoms, together with eye symptoms and throat symptoms. General symptoms usually include headache, tiredness and nausea. In this study we have chosen to study different types of weekly mucosal and general symptoms since we have the hypothesis that different types of mucosal and general symptoms can have different risk factors in the indoor environment.

The aim of the study was to investigate associations between rhinitis and other types of weekly SBS symptoms among junior high school students in Johor Bahru, Malaysia and levels of cat allergen (Fel d 1), two detected mycotoxins (verucarrol and sterigmatocysin) and five sequences of fungal DNA in the pupils’ classrooms. Verucarrol and sterigmatocysin were included in this study because they were the only mycotoxins detected in more than one classroom. Verucarrol is a mycotoxins produced by *S*. *chartarum* with toxic effects in mouse macrophages [28]. Sterigmatocystin is a mycotoxin produced mainly by *A*. *versicolor*. Spores from this mould can induce inflammation in mouse macrophages [29] and sterigmatocystin can cause inflammation in mouse lung [30]. Due to limited statistical power we have selected the most common types of mucosal symptoms (eye symptom, rhinitis, throat symptoms) and general symptoms (headache and tiredness). However, since dermal symptoms were relatively uncommon we combined them to one variable (any weekly dermal symptom). Dust samples were collected in two ways; by swabbing the top frame of the black board with a cotton swab (swab samples) and by collecting airborne settling dust with Petri dishes (Petri dish samples). We have previously published data from this study on associations between asthmatic symptoms, respiratory infections and self-reported atopy and the same types of indoor exposures in these schools [31].

## Material and Methods

### Study population

Eight schools were randomly selected from the secondary schools in Johor Bahru, Malaysia. For each selected school, four classes of grade two students were randomly selected. Finally, and 15 students in each class were randomly selected. A total of 462 students participated (96%), 223 males and 238 females. The study proposal was approved by the Medical Research and Ethics Committee of the National University of Malaysia and all participants gave informed consent. We obtained written consent from the students after explaining to them the purpose of the study and their role in answering the questionnaires. The records of study respondents’ signatures are kept at the National University of Malaysia. The students brought the questionnaire home to answer it together with their parents or guardians, but we got signatures only from the students. The study was performed in 2007 and the Ethical Committee approved this procedure since the study only included questionnaire data and no medical examinations or clinical tests. The questionnaire study and collection of dust from the schools had permission from Johor State Health Department, the principal of each school and the head teacher of each class involved in the study.

### Assessment of health data

We used a self-administered questionnaire which had previously been previously used in Swedish, Chinese and Korean school studies [28, 32, 33]. It contained questions on age, current smoking, doctors’ diagnosed asthma, allergy to cat, dog and pollen and parental allergy/asthma. SBS symptoms during the preceding 3 months included eye irritation, nasal catarrh or nasal congestion (combined to rhinitis), dry throat or sore throat (combined to throat symptoms), headache, tiredness and facial and hands rashes or itching, eczema (five dermal questions combined to dermal symptoms). Each question had four alternative answers: Yes, everyday; Yes, 1–4 times per week; Yes, 1–3 times per month; and No, never. The questionnaire was distributed to the selected students the same week as the environmental measurements and answered with help of the parents at home. Then a school-nurse went through the questionnaires during a face-to-face interview with each student to clarify any ambiguity in the questions. When answering the questionnaire, the student had no information on the data being collected from the classrooms.

### Building inspection and indoor climate

Details on construction, building materials and age, type of ventilation and heating system, and signs of dampness or mould growth were noted. Temperature (°C), Relative air humidity (RH, %) and concentration of CO_2_ (ppm) were measured in the classrooms during normal activities within 50–70 min with Q-TrakTM IAQ monitors (TSI Incorporated, St. Paul, Minnesota, USA), by logging average values over one minute. The instruments were regularly calibrated.

### Dust collection

Settled airborne dust was collected by both cotton swab and Petri dish sampling as previously described [31]. Settled dust samples were collected by swabbing 60 cm^2^ of surface (1×60 cm) from the top frame of the blackboard in each classroom. The blackboard top frame was divided into a left and right part, with the left side dust samples used for fungal DNA analysis and the right side samples for mycotoxin analysis. For fungal DNA [31] and allergen analysis [34], settled airborne dust was collected using two Petri dishes in each classroom, placed on top of open bookshelves or similar areas (height about 1.5–2.0 m) and kept open for 7 days.

### Allergens analysis

Enzyme-Linked Immunosorbent Assay (ELISA) was applied to determine the allergen levels of cat (Fel d1), dog (Can f1) (Indoor Biotechnologies Ltd, Manchester, UK), and horse (Equ c x) allergens (Mabtech, Stockholm, Sweden) using monoclonal antibodies, as previously described [32]. Amplified ELISA was used for cat allergen analysis for cases when when the allergen levels were lower than 1.0 ng/ml by conventional ELISA [32].

### Analysis of fungal DNA by qPCR

The method has been previously described [31, 35]. The cotton swab was cut into a 2 ml tube and diluted with 120 μl digestion buffer and 5μl Zymolyase. Each Petri dish was washed with 10 ml of ddH_2_O. Fungal DNA was extracted from the samples and five multiplex reactions were performed in five separate tubes targeting the DNA of the following species: total fungi, *Aspergillus spp* and *Penicillium spp* (Asp/Pen), *Aspergillus versicolor*, *Stachybotrys chartarum* and *Streptomyces sp*. The reaction targeting *A*. *versicolor* simultaneously amplified an internal positive control that was used to detect PCR inhibition. DNA was extracted from the samples using the YeaStar^™^ Genomic DNA Kit (Zymo Research, Orange, CA) according to the manufacturer’s instructions. The oligonucleotides used for amplification and detection were designed using the design software Primer Express 2.0 (Applied Biosystems, Foster City, CA USA). Primers and probes for total fungal DNA, *A*. *versicolor* and *S*. *chartarum* DNA are in the region of internal transcribed spacer 1, 5.8 S rRNA and internal transcribed spacer 2. Primers and probes for Asp/Pen DNA are in the gene for 28S rRNA and for *Strepto myces* DNA in the gene for 16S rRNA. GenBank accession numbers AB030916.1, AB002079.1, AJ639854.1, U00970.1, AF548081.1 and AF548082.1 were used to design the test for total fungal DNA: GenBank accession numbers AF454157.1, Z48340.1, DQ914661.1, AF027863.1, AF433079.1, DQ123641.1, U29632.1, AF0344456.1, AF034461.1, AF034455.1, AF033395.1 and U29555.1 were used to design the test for *Asp/Pen* DNA. GenBank accession number AJ937749.1 was used to design the test for *A*. *versicolor* DNA: GenBank accession number AY180260.1 was used to design the test for S. *chartarum* DNA: GenBank accession numbers EF017715.1 and AL939114.1 were used to design the test for *Strepromyces* DNA: Amplification and detection was performed on a 7300 Real-time PCR Instrument (Applied Biosystems, Foster City, CA USA) using the Taqman^®^ Universal Master Mix (Applied Biosystems, Foster City, CA USA). The fungal DNA levels were expressed as cell equivalents (CE), assuming one sequence per cell. The final result was presented as CE/m^2^ for swab samples and CE/m^2^/day for Petri dish samples.

Information on species captured by the different fungal DNA sequences is available in the appendix (S1 Appendix). The total fungal DNA sequence was common for 7 *Acremonium sp*., 61 *Alternaria sp*. (including *A*. *alternata*), 86 *Aspergillus sp*. (including *A*. *fumigatus* but not *A*. *versicolor*), *Aureobasidium mansonii*, *Aurerobasidium pullulans*, *Cerebella andropogonis*, 38 *Cladosporium sp*. (including *S*. *herbarium)*, 14 *Curvularia sp*., *Cylindorcarpon lichenicola*, 3 *Davidiella sp*., *Epicoccum nigrum*, 27 *Eupenicillium sp*., 6 *Eurotium sp*., 8 *Fusarium sp*., *Hemicarpenteles paradoxus*, *Mycosphaerella macrospora*, *Mycosphaerella tassiana*, *Nectria haematococca*, 17 *Neosartorya sp*. 15 *Paecillomyces sp*., 157 *Penicillium sp*., 3 *Petromyces sp*., *Ramichloridium mackenziei*, 9 *Rhinocladiella sp*, *Sclerocleista ornata*, 12 *Stachybotrys sp*., 3 *Thermoascus sp*. and 48 *Trichoderma sp*. We have used the term “total fungal DNA” for this sequence since it coverers a wide range of indoor fungi, mainly *Ascomycetes*, but it does not cover all indoor fungi. The *Asp/Pen* DNA sequence was common for 37 *Aspergillus sp*. (including *A*. *fumigatus* but not *A*. *versicolor*), *Davidiella tassiana*, 14 *Eupenicillium sp*., 15 *Eurotium sp*., *Hemicarpenteles paradoxus*, 7 *Neosartorya sp*., *Paecilomyces variotii*, *Paracoccidioides cerebriformis*, 62 *Penicillium sp*. and *Thermoascus aurantiacus*. The *Streptomyces* DNA sequence was common for 187 *Streptomyces sp*., as well as *Micromonospora megalomicea (for details see* S1 Appendix*)*. The *A*. *versicolor* DNA sequence was specific for *A*. *versicolor* only. The *S*. *chartarum* DNA sequence was specific for *Stachybotrys charatarum* and *Stachybotrys chlorohalonata* but not *Memnoninella echinata*.

### Mycotoxins analysis

The method for mycotoxin analysis has been previously described [19, 21]. The swabs were covered with methanol and extracted at room temperature overnight. After further preparation, samples were analysed by high pressure liquid chromatography (HPLC) with a tandem mass spectrometric detector (HPLC-MSMS) using reserpine as internal standard. Aflatoxin B, gliotoxin, satratoxin G, satratoxin H and sterigmatocystin were analysed by a proStar HPLC/1200L triple quadropole MSMS system (Varian Inc. Walnut Ctreek, CA, USA). Derivatives of trichodermol and verrucarol were analysed by tandem gas chromatography-mass spectrometry (GC-MSMS) using a CP-3800 GC triple quadropole MSMS system (Varian Inc.) details are provided elsewhere [19, 21]. The detection limits per sample for trichodermol and verrucarol were 6 pg, for sterigmatocystin was 12 pg, for aflatoxin B and gliotoxin were 125 pg, respectively. Finally, the amount of each mycotoxin from the swab samples was calculated and expressed as pg/m^2^.

### Statistical methods

The differences between groups were tested by the chi-square test. Differences in fungal DNA levels between classrooms with and without dampness/mould were tested by Mann-Whitney U-test. Correlations between indoor exposures were analysed by a rank correlation test (Kendal-Tau beta). Within and between school buildings variability were evaluated using linear mixed models with a random intercept on the school level, using STATA version 13.0. In this analysis, data on total fungal DNA and allergens were log-transformed. In addition, so called ‘fold ranges’, within- and between buildings (_w_R_0.95_ and _b_R_0.95_) were calculated from the variance components. The fold ranges should be interpreted as the ratio of the 97.5^th^ and 2.5^th^ percentiles of the log-normally distributed exposure [36]. As an example: A _w_R_0.95_ of 3 means that 95% of the mean value within each school can vary with a factor of 3. A _b_R_0.95_ of 3 indicates that 95% of the mean values for the schools are within a range of factor 3.

Associations between allergens, fungal DNA, mycotoxin exposure and symptoms were examined by multi-level multiple logistic regression, controlling for sex, race, smoking, atopy and parental asthma/allergy. Two levels were used, individual level and classroom level. Some models did not work when using a 3-level model (individual, classroom, school), but since similar results were obtained in 3-levels and 2-level models when both types of models could be used, we choose to report data for 2-levels models only. When analysing association for dermal symptoms, smoking could not be included in the models due to small numbers in some cells. For the same reasons, associations between sterigmatocystin and tiredness could not be analysed. Finally, we analysed associations between risk factors (personal factors and school exposure) and symptoms by stepwise multiple logistic regression (forward Wald). Significant factors in the stepwise regression models were then entered in 2-level logistic regression models. Odds ratio (OR) with 95% confidence interval (95% CI) was calculated for the logistic regression analyses. Statistics were performed with the Statistical Package for the Social Sciences (SPSS) 21.0 or the STATA statistical package version 13 (for multi-level logistic regression and linear mixed models), using two-tailed tests and a 5% significance level.

## Results

A total of 52% were girls and mean age was 14 years (range 14–16). The prevalence of doctors’ diagnosed asthma was high (13.1%), and 21.1% reported pollen or furry pet allergy (atopy). The prevalence of smoking was 4.8% in boys and 1.3% in girls (p<0.001), 42.3% were Malays, 42.7% Chinese and 15.0% Indian. SBS symptoms were common, especially mucosal and general symptoms. Girls had more often headache and tiredness (Table 1).

**Table 1:** Prevalence of weekly symptoms of the last 3 months among students from junior high schools in Johor Bahru, Malaysia (N = 362).

Symptoms	Overall (%)	Male (%)	Female (%)	*P*-value
**Any ocular symptom**^a^	11.6	12.8	10.5	0.44
Runny nose	11.5	10.9	12.1	
Nasal congestion	14.9	16.5	13.4	
**Any nasal symptom**^b^ **(rhinitis)**	18.8	19.3	18.3	0.79
Dryness in the throat	10.7	8.3	13.1	
Sore throat	10.4	9.7	10.9	
**Any throat symptom**^c^	15.6	13.0	18.1	0.14
Rash in the face	0.5	0	0.9	
Itch in the face	2.2	2.3	2.1	
Rash on the hands	3.1	2.7	3.4	
Itch on the hands	7.6	4.6	10.4	
**Any dermal symptom**^d^	11.1	8.2	13.8	0.06
**Headache**	20.6	16.4	24.6	0.03
**Tiredness**	22.1	16.6	27.4	0.006

^a^Dry eyes or itching in the eyes (one question)

^b^The prevalence of students with at least one symptom (runny nose or nasal congestion) classified as nasal

^c^The prevalence of students with at least one symptom (dryness in the throat or sore throat) classified as throat

^d^The prevalence of students with at least one symptom (facial and hand rash or itching) classified as dermal

The mean age of the school buildings was 16 years (range 3–40). They were 2–4 storeys concrete buildings with painted indoor surfaces and the floor surface consisted of concrete without any paint or floor covering. There were no carpets, pot plants or book shelves in any classroom. All classrooms had electric fans in the ceiling; none had an air conditioning unit or a mechanical ventilation system. All classrooms were equipped with venetian blinds on both sides, enabling the outdoor air to enter though one window and exit through the other. All classrooms hade closed doors and open windows during the lectures. The mean room temperature was 29°C (range 27–31), similar as the mean outdoor temperature (29°C). The mean indoor relative air humidity (RH) was 70% (range 60–78%), similar as the mean outdoor air humidity (73%). The mean CO_2_ concentration in the classrooms was 490 ppm (range 380–690 ppm) and 410 ppm outdoors. The mean number of students per classroom was 45 (range 24–42). Five (16%) classrooms had visible signs of water leakage, but none had visible indoor mould growth.

Total fungal DNA and *Asp/Pen* DNA were detected in all classrooms both for Petri dish and swab samples. *A*. *versicolor* DNA was detected in 70% of the Petri dish samples, while *S*. *chartarum* was found in 13% and *Streptomyces* DNA in 87% of the samples. *A*. *versicolor* DNA was detected in 56% of the swab samples, while *S*. *chartarum* DNA was detected in 3% and *Streptomyces* DNA in 28% of the samples. Cat (Fel d1) allergen was detected in all Petri dish dust samples but dog or horse allergen was not detected in any. The mean number of cat owners per classroom was 2.1 (range 0–6) and mean number of dog owners per classroom was 1.8 (range 0–4). Aflatoxin B was detected in one swab sample (3%), sterigmatocystin in two samples (6%) and verrucarol in four samples (12%). Data on arithmetic mean and median levels is given in table 2. For exposures found in few samples only mean is presented. There were significant correlations between cat allergen and total fungal DNA, *Asp/Pen* DNA and *Streptomyces* DNA and between total fungal DNA, Asp/Pen DNA and *Streptomyces* DNA (Table 3). There were no significant correlations between the five types fungal DNA in swab samples (data not shown).

**Table 2:** Fungal DNA, mycotoxins and cat allergen (Fel d 1) in the classrooms (N = 32).

Fungal DNA/mycotoxins/allergen	In swab samples	In swab samples	In Petri dish samples	In Petri dish samples
	AM (SD)	Median (IQR)	AM (SD)	Median (IQR)
Total fungal DNA	7.17*10^8^ (4.91)	5.74*10^8^ (4.10–9.88)	11.07*10^6^ (7.42)	8.89*10^6^ (6.23–13.53)
*Asp/Pen* DNA	1.61*10^8^ (16.1)	0.99*10^8^ (0.44–2.52)	2.79*10^6^ (3.73)	1.35*10^6^ (0.79–4.11)
*A*. *versicolor* DNA	8780 (12174)	NA	NA	NA
*Streptomyces* DNA	893 (1634)	NA	NA	NA
Sterigmatocystin	1.72 (8.33)	NA	NA	NA
Verrucarol	2.55 (9.58)	NA	NA	NA
Cat allergen	NA	NA	8.99 (6.60)	7.77 (3.29–13.0)

AM = arithmetic mean, SD = Standard deviation, IQR = Interquartile range, CE = cell equivalents NA = not available

*Asp/Pen*: *Aspergillus*/*Penicillium*, *A*. *versicolor*: *Aspergillus versicolor*

Total fungal DNA, *Asp/Pen* DNA, *A*. *versicolor* DNA and *Streptomyces* DNA were expressed as CE/m^2^ in swab samples and as CE/m^2^ per day in Petri dish samples

Sterigmatocystin and verrucarol in swab samples were expressed as ng/m^2^

Fel d 1 was expressed as ng/m^2^ per day in Petri dish samples

**Table 3:** Correlation (Kendal Tau beta) between fungal DNA and cat allergen (Fel d 1) in Petri dish samples from the classrooms (N = 32).

Type of fungal DNA/allergen	Total Fungal DNA	*Asp/Pen* DNA	*A*. *versicolor* DNA	*S*. *chartarum* DNA	*Streptomyces* DNA	Cat allergen
Total fungal DNA	1					
*Asp/Pen* DNA	0.46***	1				
*A*. *versicolor* DNA	0.43***	0.28*	1			
*S*. *chartarum* DNA	-0.06	0.02	-0.08	1		
*Streptomyces* DNA	0.30*	0.39**	0.24	-0.13	1	
Cat allergen	0.29*	0.41**	0.22	-0.08	0.35**	1

*p<0.05

**p<0.01

***p<0.001

For total fungal DNA, there was less variation between buildings than within buildings for both sampling methods. For example: the mean values between the schools for total fungal DNA in swab dust samples (_b_R_0.95_ = 6) indicated that 95% of the mean values between the schools for total fungal DNA were within a range of factor 6. In contrast, A _w_R_0.95_ of 9 means that 95% of the mean value within each school for total fungal DNA in swab dust samples could vary with a factor of 9. For *Asp/Pen* DNA and cat allergen, there was more variation between buildings than within buildings (Table 4).

**Table 4:** Variation within and between buildings, and fold ranges, of total fungal DNA and cat allergen (Fel d 1) in eight secondary school buildings (32 classrooms) in Johor Bahru, Malaysia.

Fungal DNA/Allergen	Type of sample	Variation within building (%)	Variations between buildings (%)	_w_R_0.95_^a^	_b_R_0.95_^a^
Total fungal DNA	In swab samples	61	39	9	6
	In Petri dish samples	56	44	7	5
*Asp/Pen* DNA	In swab samples	28	72	12	59
	In Petri dish samples	44	56	17	25
Cat allergen	In Petri dish samples	35	65	14	38

^a^Variance ratios (‘‘fold-ranges”) within (w) and between buildings (b), calculated from the variance components of the 97.5^th^ and 2.5^th^ percentiles of the log-normally distributed exposure.

Initially, we analysed associations between symptoms and classroom exposure, controlling for gender, race, tobacco smoking, atopy and parental asthma/allergy. There were positive associations between total fungal DNA in swab samples and ocular symptoms (p = 0.03), rhinitis (p = 0.006), throat symptoms (p = 0.04) and tiredness (p = 0.01). There was a negative (protective) association between *Streptomyces* DNA and rhinitis (p = 0.04) (Table 5). For Petri dish samples, only two significant associations were found. There was a positive association between *S*. *chartarum* DNA and dermal symptoms (OR = 1.15; 95% CI 1.03–1.29; p = 0.01) expressed for an increase of 100 CE/m^2^ per day. Moreover, there was an association between cat allergen levels (Der f 1) and headache (OR = 1.77; 95% CI 1.17–2.68; p = 0.007) expressed for an increase of 10 ng/m^2^ per day.

**Table 5:** Associations between levels of fungal DNA and mycotoxins in swab samples from the classrooms and weekly symptoms among the students (N = 462).

Fungal DNA/ mycotoxins	Any ocular symptom	Any nasal symptom (rhinitis)	Any throat symptom	Any dermal symptom	Headache	Tiredness
Total fungal DNA	3.12 (1.12–8.70)*	2.96 (1.37–6.42)**	2.56 (1.03–6.34)*	0.51 (0.19–1.38)	1.57 (0.75–3.30)	3.60 (1.31–9.94)*
*Asp/Pen* DNA	0.94 (0.71–1.24)	0.91 (0.74–1.13)	1.05 (0.83–1.33)	0.97 (0.72–1.30)	0.88 (0.71–1.08)	0.83 (0.63–1.10)
*A*. *versicolor* DNA	0.85 (0.56–1.28)	0.88 (0.66–1.17)	0.89 (0.64–1.24)	0.97 (0.66–1.43)	0.91 (0.69–1.19)	0.98 (0.68–1.41)
*Streptomyces* DNA	0.80 (0.57–1.13)	0.76 (0.58–0.99)*	0.80 (0.59–1.08)	1.15 (0.86–1.52)	0.94 (0.73–1.20)	0.73 (0.52–1.01)
Sterigmato-cystin	0.93 (0.52–1.68)	0.26 (0.02–3.33)	0.79 (0.44–1.43)	NA	1.14 (0.78–1.67)	NA
Verrucarol	1.20 (0.75–1.93)	1.36 (0.96–1.92)	1.21 (0.77–1.91)	0.77 (0.42–1.40)	0.74 (0.43–1.29)	1.05 (0.64–1.74)

Reported data are Odds Ratio (OR) with 95% Confidence Interval (CI) by a 2-level hierarchic logistic regression model adjusted for gender, ethnicity, current smoking, atopy and heredity.

NA Not analysed (too few numbers with a combination of symptoms and exposure)

(OR calculated for 10^9^ CE/m^2^ increase in total fungal DNA)

(OR calculated for 10^8^ CE/m^2^ increase in *Asp/Pen* DNA)

(OR calculated for 10^4^ CE/m^2^ increase in *A*. *versicolor* DNA)

(OR calculated for 100 CE/m^2^ increase in *Streptomyces* DNA)

(OR calculated for 10 ng/m^2^ increase in sterigmatocystin)

(OR calculated for 10 ng/m^2^ increase in verrucarol)

*p<0.05

**p<0.01

As a next step we applied stepwise logistic regression analysis (forward, Wald) to select significant variables in six combined models (one for each symptom). These variables were then entered in six 2-level logistic regression models (pupil and classroom level). Parental asthma/allergy (p<0.001), total fungal DNA in Swab samples (p = 0.009) and *A*. *versicolor* DNA in Petri dish samples (p = 0.02) were associated with ocular symptoms. Parental asthma/allergy (p = 0.001) and total fungal DNA in swab samples (p = 0.02) were associated with rhinitis. Throat symptoms were associated with atopy (p = 0.001). Headache was associated with atopy (p = 0.007) and Chinese students had less headache (p = 0.005). Girls had more tiredness (p = 0.004) and there were positive associations between tiredness and total fungal DNA in swab samples (p = 0.001), *A*. *versicolor* DNA in Petri dish samples (p = 0.001) and verrucarol in swab samples (p = 0.03). Moreover, there were negative associations between tiredness and *Streptomyces* DNA is swab samples (p = 0.03) and in Petri dish samples (p = 0.03) (Table 6).

**Table 6:** Final models (2-level hierachic logistic regression) for associations between fungal DNA and mycotoxins in the classrooms and weekly symptoms among the students (N = 462).

	OR (95% CI)	*P*-value
**Any ocular symptom**		
Parental asthma/allergy	3.64 (1.85–7.19)	<0.001
Total fungal DNA in swab samples	3.43 (1.36–8.62)	0.009
*Streptomyces* DNA in swab samples	0.79 (0.58–1.08)	0.14
*A*. *versicolor* DNA in Petri dish samples	4.81 (1.36–17.0)	0.02
**Any nasal symptom (rhinitis)**		
Chinese	0.99 (0.52–1.89)	0.97
Indian	0.39 (0.15–1.05)	0.06
Atopy	1.88 (0.98–3.59)	0.06
Parental asthma/allergy	3.75 (2.06–6.85)	<0.001
Total fungal DNA in swab samples	2.16 (1.12–4.17)	0.02
**Any throat symptom**		
Current smoker	2.44 (0.86–6.94)	0.09
Atopy	2.81 (1.55–5.11)	0.001
*Asp/Pen* DNA swab samples	1.21 (0.99–1.47)	0.06
**Any dermal symptom**		
*S*. *Chartarum* DNA Petri dish	1.09 (0.99–1.47)	0.06
**Headache**		
Female	1.46 (0.88–2.42)	0.14
Chinese	0.43 (0.24–0.77)	0.005
Indian	0.77 (0.39–1.55)	0.47
Atopy	2.13 (1.23–3.69)	0.007
**Tiredness**		
Female	2.26 (1.30–3.94)	0.004
Chinese	1.13 (0.60–2.13)	0.71
Indian	0.40 (0.15–1.10)	0.08
Parental asthma/allergy	1.68 (0.92–3.06)	0.09
Total fungal DNA in swab samples	3.72 (1.74–7.94)	0.001
*Streptomyces* DNA in swab samples	0.74 (0.57–0.97)	0.03
Verrucarol in swab samples	1.60 (1.02–2.51)	0.04
*A*. *versicolor* DNA in Petri dish samples	7.19 (2.17–23.8)	0.001
*Streptomyces* DNA in Petri dish samples	0.62 (0.45–0.85)	0.03

The models include variables retained in a stepwise logistic regression model (forward regression Wald statistics, p<0.10). These variables were entered in a 2-level hierarchic logistic regression model adjusting for classroom level.

(OR calculated for 10^9^ CE/m^2^ increase in total fungal DNA in swab samples)

(OR calculated for 10^8^ CE/m^2^ increase in *Asp/Pen* DNA in swab samples)

(OR calculated for 100 CE/m^2^ increase in *Streptomyces* DNA in swab samples)

(OR calculated for 10 ng/ m^2^ increase in verrucarol in swab samples)

(OR calculated for 1000 CE/m^2^ per day increase in *A*. *versicolor* DNA in Petri dish samples)

(OR calculated for 1000 CE/m^2^ per day increase in *Streptomyces* DNA in Petri dish samples)

Total fungal DNA and *Streptomyces* DNA in swab samples and *A*. *versicolor* DNA and *Streptomyces* DNA in Petri dish samples were significantly associated, either positively or negatively, with rhinitis or SBS symptoms. We compared levels of these four fungal DNA sequences between classrooms with (N = 5) and without signs of dampness (N = 27) using Mann-Whitney U-test. The level of total fungal DNA in swab samples were two times higher in classrooms with signs of dampness; mean level was 13.03*10^8^ CE/m^2^ in rooms with dampness and 6.08*10^8^ CE/m^2^ in rooms without dampness (p = 0.02). The other three DNA sequences were not associated with signs of dampness in the classrooms.

## Discussion

Rhinitis and SBS symptoms, particularly headache and tiredness, were common among secondary school students in Johor Bahru, Malaysia. Total fungal DNA and the mycotoxin verrucarol in swab samples and *Aspergillus versicolor* DNA in Petri dish samples were positively associated with rhinitis and SBS symptoms.

In this study, schools, classrooms and students were randomly selected from among all secondary schools in Johor Bahru and the response rate was high. All samples were analysed after questionnaire data collection was completed, and environmental sampling was conducted the same week as the questionnaire study. Johor Bahru has a tropical climate with similar outdoor climate all year around; so the fact that the study was performed in November does not influence the outcome as the ventilation characteristics and both indoor and outdoor exposure parameters would be expected to be constant over the year. Thus we conclude that the study was not seriously influenced by selection or information bias.

More girls reported tiredness compared to boys and Chinese students reported less tiredness compared to Malay or Indian students. Other school studies from Asia have found different results concerning gender differences in SBS symptoms. In two previous SBS studies in junior high school students in Taiyuan, China, no gender differences were observed for general SBS symptoms [27, 37] and in one of these studies, boys reported more dermal and mucosal symptoms than girls [27]. In another study on SBS symptoms among elementary school students in Hokkaido, Japan, boys reported more SBS than girls, especially nasal symptoms [38]. In one study on headache and tiredness in elementary school students in Korea, girls had more headache but they found no gender difference for tiredness [25]. Finally, one study from Denmark on SBS symptoms among adolescent school children found that girls reported more of most types of symptoms, including eye and dermal symptoms, headache and dizziness [39]. Thus, gender differences in SBS symptoms can depend on age as well as cultural differences.

The level of CO_2_ was low in the classrooms (range 380–690 ppm) and always below the recommended limit of 1000 ppm [40]. This was due to effective natural ventilation since all classrooms had windows with venetian blinds on both sides of the classrooms. Despite the high ventilation flow, fungal DNA was common in the schools and associated with SBS symptoms. The most consistent associations were found between total fungal DNA in swab samples and SBS symptoms, especially for ocular symptoms, rhinitis and tiredness. To our knowledge, there are very few studies on associations between rhinitis or SBS-symptoms and fungal DNA in schools. In one multi-centre study from primary schools in five countries in Europe, levels of total fungal DNA in vacuumed dust was associated with rhinitis especially when comparing the highest and lowest tertiles of exposure [41]. In a longitudinal study on SBS-symptoms among junior high school students in Taiyuan, China, total level of fungal DNA in Petri dish samples from the classrooms were associated with a higher incidence of school-related SBS-symptoms as well as a higher remission of mucosal symptoms [26].

In our final regression models, we found an association between levels of *A*. *versicolor* DNA in Petri dish samples and tiredness. There are few epidemiological studies on associations between fungal exposure and headache and tiredness. In one previous case study in a water-damaged building, where moderate to high levels of fungi were measured (*Penicillium spp*., *A*. *versicolor*, *S*. *chartarum*), occupants reported mainly neurobehavioral and upper respiratory tract symptoms, and symptoms were less prevalent after relocation from the water-damaged building [42]. In one French study, a subgroup of office workers who handled mouldy documents reported more headache and fatigue. In this study, *Penicillium chrysogenum*, *Cladosporium sphaerospermum*, and *A*. *versicolor* were the three main fungi in air and dust in terms of quantity and frequency [43].

Verrucarol was found in four of the 32 classrooms (12%) and students in these classrooms reported more tiredness. To our knowledge, this is the first study on associations between mycotoxins in schools and SBS symptoms. However, descriptive data has been published for a large number of mycotoxins, called secondary fungal metabolites, in European schools in Spain, the Netherlands and Finland in the HITEA study. Many types of mycotoxins were detected in school dust, but verrucarol was one of the least common mycotoxins and was detected only in a few percent of the schools [18]. There are very few epidemiological studies on associations between mycotoxins in indoor environments and occupants health. One case-control study measured mycotoxins in urine of patients with chronic fatigue syndrome and compared levels with healthy controls. They found significantly higher levels of mycotoxins, including the monocyclic trichothenes, in cases as compared to controls. Moreover, environmental testing in the home in a subset if the patients confirmed mould and mycotoxin exposure [44]. In our study we found an association between verrucarol and tiredness, but not between *S*. *chartarum* DNA and tiredness. This can be because some of the species detected by the *S*. *Stachybotrys* DNA test does not produce verrucarol. Verrucarol is produced by S. *chartarum* [45] but not by *Memnoninella echinata* [45]. It is well known that in subtropical environments, *Memnoninella echinata* replaces the two *Stachybotrys* species [46].

We found consistent negative associations between *Streptomyces* DNA (both in swab and Petri dish dust samples) and tiredness. *Streptomyces spp*. is a large genus of Gram-positive bacteria, and some species have been shown to produce inflammatory reactions in vitro and in vivo [28]. Moreover, *Streptomyces* can produce geosmin, a microbial volatile organic compound MVOC) with a soil odour [47]. A study from USA concluded that indoor *Streptomyces* DNA may have mostly outdoor sources [48]. We found no previous epidemiological studies on *Streptomyces* in indoor environments and SBS symptoms. However, one Chinese school environment study found that the remission of general SBS symptoms were higher at higher levels of muramic acid, a cell wall compound mainly found in gram positive bacteria [26]. The negative association between *Streptomyces* DNA and tiredness in our study remains unclear and needs to be confirmed in other studies.

In this study we used swab samples and Petri dish samples to collect dust for analysis of allergens and fungal DNA. These sampling methods have certain limitations. Pumped air sampling over a longer period (days or weeks) would be the best method (gold standard) to assess exposure to particle pollutants in indoor environments but can be difficult to apply in epidemiological studies. The main limitation with swab samples is that they cover only a smaller fraction of the total area in the room, and moreover we do not know it the particles collects by the swabs have been airborne. Settled airborne dust, collected by Petri dishes, has been suggested as a suitable surrogate for airborne exposure in studies that explore indoor microbes [49]. Petri dish sampling is influenced by the activity in the room causing resuspension of dust from indoor surfaces (e.g. floors) to the air [50]. In classrooms, it has been shown that the concentration of larger particles increase when the room is occupied and the increase of larger particles is responsible for elevation of bacterial markers in the air during occupation [51]. Since our study included bot occupied and non-occupied periods and sampled settling airborne dust for about one week in all classrooms, we consider Petri dish sampling as a reasonable proxy variable for airborne exposure in the classrooms.

In conclusion, fungal DNA and cat allergen were common in the studied Malaysian schools and there was high prevalence of rhinitis and SBS-symptoms among the students, especially headache and tiredness. Total fungal DNA in the classrooms can be associated with SBS symptoms such as ocular symptoms, rhinitis and tiredness. The positive associations for species specific sequences (*A*. *versicolor* and *Streptomyces sp*.) illustrate the importance of analysing microbial exposure on species level when studying associations with SBS symptoms. Analysis of fungal DNA by qPCR in dust collected by swab and Petri dish sampling seems to be a promising method to monitor mould exposure in schools. The advantage with qPCR is that it can detect both general and specific DNA sequences irrespective of whether the organisms are viable or non-viable. Verrucarol, a mycotoxin produced by *S*. *chartarum*, could be a risk factor for tiredness. Our study indicates that mycotoxins could be common in indoor environments in tropical areas. As tropical areas are expected to have high microbial exposure because of the hot and humid climate, more studies are needed on health effects of indoor exposure to mould in the tropics. From a public health perspective, further epidemiological studies identifying and quantifying specific fungal species and mycotoxins in schools in different climate zones are needed.

## Supporting Information

S1 Appendix Species detected by the total fungal DNA test, the *Asp/Pen* DNA test and the *Streptomyces* DNA test. (DOC)
