# Supplementary material for: Rhinitis, Ocular, Throat and Dermal Symptoms, Headache and Tiredness among Students in Schools from Johor Bahru, Malaysia: Associations with Fungal DNA and Mycotoxins in Classroom Dust
Source: PLoS One. 2016 Feb 1;11(2):e0147996. doi: 10.1371/journal.pone.0147996 (PMC4734676; doi:10.1371/journal.pone.0147996)
Supplement: S1 Appendix — (DOC) [file pone.0147996.s001.doc]

**S1 Appendix Species detected by the total fungal DNA test, the *Asp/Pen* DNA test and the *Streptomyces* DNA test.**

**Species detected by the total fungal DNA test**

| *Acremonium butyri* |
| --- |
| *Acremonium chrysogenum* |
| *Acremonium crotocinigenum* |
| *Acremonium implicatum* |
| *Acremonium kiliense* |
| *Acremonium strictum* |
| *Acremonium zeae* |
| *Alternaria abutilonis* |
| *Alternaria aff. dianthicola* |
| *Alternaria aff. longipes* |
| *Alternaria alli isolate* |
| *Alternaria alternata* |
| *Alternaria arborescens* |
| *Alternaria arbusti* |
| *Alternaria astragali* |
| *Alternaria brassicae* |
| *Alternaria brassicicola* |
| *Alternaria carotiincultae* |
| *Alternaria carthami* |
| *Alternaria cheiranthi* |
| *Alternaria cichorii* |
| *Alternaria cinerariae* |
| *Alternaria citri* |
| *Alternaria compacta* |
| *Alternaria conjuncta* |
| *Alternaria crassa* |
| *Alternaria daturicola* |
| *Alternaria dauci* |
| *Alternaria destruens* |
| *Alternaria dianthi* |
| *Alternaria dianthicola* |
| *Alternaria eichhorniae* |
| *Alternaria gaisen* |
| *Alternaria helianthi* |
| *Alternaria japonica* |
| *Alternaria jesenskae* |
| *Alternaria leucanthemi* |
| *Alternaria lini* |
| *Alternaria linicola* |
| *Alternaria longipes* |
| *Alternaria longissima* |
| *Alternaria macrospora* |
| *Alternaria mali* |
| *Alternaria metachromatica* |
| *Alternaria multirostrata* |
| *Alternaria novae-zelandiae* |
| *Alternaria oregonensis* |
| *Alternaria palandui* |
| *Alternaria panax* |
| *Alternaria passiflorae* |
| *Alternaria petroselini* |
| *Alternaria pomicola* |
| *Alternaria porri* |
| *Alternaria protenta* |
| *Alternaria radicina* |
| *Alternaria raphani* |
| *Alternaria seleniiphila* |
| *Alternaria selini* |
| *Alternaria sesamicola* |
| *Alternaria smyrnii* |
| *Alternaria solani* |
| *Alternaria tagetica* |
| *Alternaria tenuissima* |
| *Alternaria thalictrigena* |
| *Alternaria tomatophila* |
| *Alternaria triticimaculans* |
| *Alternaria triticina* |
| *Alternaria zinniae* |
| *Aspergillus aculeatus* |
| *Aspergillus allahabadii* |
| *Aspergillus arvii* |
| *Aspergillus auricomus* |
| *Aspergillus awamori* |
| *Aspergillus avenaceus* |
| *Aspergillus bombycis* |
| *Aspergillus brasiliensis* |
| *Aspergillus brevipes* |
| *Aspergillus bridgeri* |
| *Aspergillus caelatus* |
| *Aspergillus caesiellus* |
| *Aspergillus campestris* |
| *Aspergillus candidus* |
| *Aspergillus carbonarius* |
| *Aspergillus carneus* |
| *Aspergillus cervinus* |
| *Aspergillus cf. flavipes* |
| *Aspergillus cf. niger* |
| *Aspergillus cf. Tubingensis* |
| *Aspergillus clavatoflavus* |
| *Aspergillus clavatonanicus* |
| *Aspergillus clavatus* |
| *Aspergillus costaricaensis* |
| *Aspergillus crystallinus* |
| *Aspergillus dimorphicus* |
| *Aspergillus duricaulis* |
| *Aspergillus elegans* |
| *Aspergillus ellipticus* |
| *Aspergillus flavipes* |
| *Aspergillus flavofurcatus* |
| *Aspergillus flavus* |
| *Aspergillus foetidus* |
| *Aspergillus fresenii* |
| *Aspergillus fumigatiaffinis* |
| *Aspergillus fumigatus* |
| *Aspergillus fumisynnematus* |
| *Aspergillus heteromorphus* |
| *Aspergillus homomorphus* |
| *Aspergillus ibericus* |
| *Aspergillus iizukae* |
| *Aspergillus janus* |
| *Aspergillus japonicus* |
| *Aspergillus lacticoffeatus* |
| *Aspergillus lanosus* |
| *Aspergillus lentulus* |
| *Aspergillus leporis* |
| *Aspergillus longivesica* |
| *Aspergillus malodoratus* |
| *Aspergillus melleus* |
| *Aspergillus niger* |
| *Aspergillus nomius* |
| *Aspergillus novofumigatus* |
| *Aspergillus nutans* |
| *Aspergillus ochraceus* |
| *Aspergillus oryzae* |
| *Aspergillus ostianus* |
| *Aspergillus pallidus* |
| *Aspergillus paradoxus* |
| *Aspergillus parasiticus* |
| *Aspergillus penicillioides* |
| *Aspergillus petrakii* |
| *Aspergillus phoenicis* |
| *Aspergillus piperis* |
| *Aspergillus pseudotamarii* |
| *Aspergillus restric* |
| *Aspergillus restrictus* |
| *Aspergillus ruber* |
| *Aspergillus sclerotioniger* |
| *Aspergillus sclerotiorum* |
| *Aspergillus sepultus* |
| *Aspergillus sojae* |
| *Aspergillus sulphureus* |
| *Aspergillus tamarii* |
| *Aspergillus terreus* |
| *Aspergillus terricola* |
| *Aspergillus thomii* |
| *Aspergillus togoensis* |
| *Aspergillus toxicarius* |
| *Aspergillus tubigensis* |
| *Aspergillus unilateralis* |
| *Aspergillus vadensis* |
| *Aspergillus wentii* |
| *Aspergillus westerdijkiae* |
| *Aspergillus viridinutans* |
| *Aspergillus zonatus* |
| *Aureobasidium mansonii* |
| *Aureobasidium pullulans* |
| *Cerebella andropogonis* |
| *Cladosporium adianticola strain* |
| *Cladosporium aff. Cladosporioides* |
| *Cladosporium allii-cepae* |
| *Cladosporium allii-porri* |
| *Cladosporium breviramosum* |
| *Cladosporium bruhnei* |
| *Cladosporium castellanii* |
| *Cladosporium cf. subtilissimum* |
| *Cladosporium cladosporioides* |
| *Cladosporium colocasiae* |
| *Cladosporium coralloides* |
| *Cladosporium cucumerinum* |
| *Cladosporium elatum* |
| *Cladosporium funiculosum* |
| *Cladosporium gossypiicola* |
| *Cladosporium herbaroides* |
| *Cladosporium langeronii* |
| *Cladosporium laxicapitulatum* |
| *Cladosporium lignicola* |
| *Cladosporium macrocarpum* |
| *Cladosporium magnusianum* |
| *Cladosporium malorum* |
| *Cladosporium multigeniculatum* |
| *Cladosporium oxysporium* |
| *Cladosporium porophorum* |
| *Cladosporium pseudiridis* |
| *Cladosporium ramotenellum* |
| *Cladosporium sinuosum* |
| *Cladosporium sphaerospermum* |
| *Cladosporium spinulosum* |
| *Cladosporium subinflatum* |
| *Cladosporium subtilissimum* |
| *Cladosporium tenellum* |
| *Cladosporium tenuissimum* |
| *Cladosporium uniseptosporum* |
| *Cladosporium uredinicola* |
| *Cladosporium variabile* |
| *Cladosporium vignae* |
| *Curvularia affinis* |
| *Curvularia brachyspora* |
| *Curvularia clavata* |
| *Curvularia cymbopogonis* |
| *Curvularia eragrostidis* |
| *Curvularia geniculata* |
| *Curvularia gladioli* |
| *Curvularia gudauskasii* |
| *Curvularia heteropogonicola* |
| *Curvularia inaequalis* |
| *Curvularia intermedia* |
| *Curvularia oryzae* |
| *Curvularia panici* |
| *Curvularia trifolii* |
| *Cylindrocarpon lichenicola* |
| *Davidiella dianthi* |
| *Davidiella macrospora* |
| *Davidiella tassiana* |
| *Epicoccum nigrum* |
| *Eupenicillium alutaceum* |
| *Eupenicillium anatolicum* |
| *Eupenicillium baarnense* |
| *Eupenicillium bovifimosum* |
| *Eupenicillium brefeldianum* |
| *Eupenicillium cinnamopurpureum* |
| *Eupenicillium crustaceum* |
| *Eupenicillium egyptiacum* |
| *Eupenicillium ehrlichii* |
| *Eupenicillium erubescens* |
| *Eupenicillium hirayamae* |
| *Eupenicillium inusitatum* |
| *Eupenicillium javanicum* |
| *Eupenicillium katangense* |
| *Eupenicillium lapidosum* |
| *Eupenicillium lassenii* |
| *Eupenicillium levitum* |
| *Eupenicillium meridianum* |
| *Eupenicillium parvum* |
| *Eupenicillium pinetorum* |
| *Eupenicillium reticulisporum* |
| *Eupenicillium rubidurum* |
| *Eupenicillium shearii* |
| *Eupenicillium stolkiae* |
| *Eupenicillium terrenum* |
| *Eupenicillium tropicum* |
| *Eupenicillium tularense* |
| *Eurotium amstelodami* |
| *Eurotium chevalieri* |
| *Eurotium herbariorum* |
| *Eurotium niveoglaucum* |
| *Eurotium repens* |
| *Eurotium rubrum* |
| *Fusarium ambrosium* |
| *Fusarium falciforme* |
| *Fusarium flocciferum* |
| *Fusarium lateritium* |
| *Fusarium lichenicola* |
| *Fusarium oxysporum* |
| *Fusarium solani* |
| *Fusarium tricinctum* |
| *Hemicarpenteles paradoxus* |
| *Mycosphaerella macrospora* |
| *Mycosphaerella tassiana* |
| *Nectria haematococca* |
| *Neosartorya aurata* |
| *Neosartorya aureola* |
| *Neosartorya botucatensis* |
| *Neosartorya coreana* |
| *Neosartorya fennelliae* |
| *Neosartorya fischeri* |
| *Neosartorya glabra* |
| *Neosartorya hiratsukae* |
| *Neosartorya laciniosa* |
| *Neosartorya primulina* |
| *Neosartorya pseudofischeri* |
| *Neosartorya quadricincta* |
| *Neosartorya spathulata* |
| *Neosartorya spinosa* |
| *Neosartorya stramenia* |
| *Neosartorya tatenoi* |
| *Neosartorya udagawae* |
| *Paecilomyces aerugineus* |
| *Paecilomyces anatarcticus* |
| *Paecilomyces carneus* |
| *Paecilomyces cateniobliquus* |
| *Paecilomyces cicadae* |
| *Paecilomyces lilacinus* |
| *Paecilomyces major* |
| *Paecilomyces marquandii* |
| *Paecilomyces niphetodes* |
| *Paecilomyces penicillatus* |
| *Paecilomyces reniformis* |
| *Paecilomyces sinensis* |
| *Paecilomyces variotii* |
| *Paecilomyces verticillatus* |
| *Paecilomyces viridis strain* |
| *Penicillium aculeatum* |
| *Penicillium adametzii* |
| *Penicillium adametzioides* |
| *Penicillium aethiopicum* |
| *Penicillium albocoremium* |
| *Penicillium allii* |
| *Penicillium angulare* |
| *Penicillium angularum* |
| *Penicillium asperosporum* |
| *Penicillium atramentosum* |
| *Penicillium atrovenetum* |
| *Penicillium aurantiogriseum* |
| *Penicillium aurantiovirens* |
| *Penicillium bilaiae* |
| *Penicillium biourgeianum* |
| *Penicillium boreae* |
| *Penicillium brasilianum* |
| *Penicillium brevicompactum* |
| *Penicillium brevistipitatum* |
| *Penicillium brocae* |
| *Penicillium camemberti* |
| *Penicillium canariense* |
| *Penicillium canescens* |
| *Penicillium capsulatum* |
| *Penicillium carneum* |
| *Penicillium cecidicola* |
| *Penicillium cf. Paneum* |
| *Penicillium charlesii* |
| *Penicillium chermesinum* |
| *Penicillium chrysogenum* |
| *Penicillium ciegleri* |
| *Penicillium cinerascens* |
| *Penicillium citreonigrum* |
| *Penicillium citrinum* |
| *Penicillium clavigerum* |
| *Penicillium coffeae* |
| *Penicillium commune* |
| *Penicillium concentricum* |
| *Penicillium coprobium* |
| *Penicillium coprophilum* |
| *Penicillium coralligerum* |
| *Penicillium cordubense* |
| *Penicillium corylophilum* |
| *Penicillium crustosum* |
| *Penicillium cyaneum* |
| *Penicillium cyclopium* |
| *Penicillium daleae* |
| *Penicillium decaturense* |
| *Penicillium decumbens* |
| *Penicillium dendriticum* |
| *Penicillium digitatum* |
| *Penicillium dipodomyicola* |
| *Penicillium dipodomyis* |
| *Penicillium discolor* |
| *Penicillium diversum* |
| *Penicillium donkii* |
| *Penicillium dravuni* |
| *Penicillium duclauxii* |
| *Penicillium echinulatum* |
| *Penicillium expansum* |
| *Penicillium fagi* |
| *Penicillium farinosum* |
| *Penicillium fellutanum* |
| *Penicillium freii* |
| *Penicillium funiculosum* |
| *Penicillium fuscum* |
| *Penicillium glabrum* |
| *Penicillium gladioli* |
| *Penicillium glandicola* |
| *Penicillium granulatum* |
| *Penicillium griseofulvum* |
| *Penicillium griseoroseum* |
| *Penicillium herquei* |
| *Penicillium hirsutum* |
| *Penicillium hordei* |
| *Penicillium implicatum* |
| *Penicillium indicum* |
| *Penicillium isariiforme* |
| *Penicillium islandicum* |
| *Penicillium italicum* |
| *Penicillium jamesonlandense* |
| *Penicillium janczewski* |
| *Penicillium janthinellum* |
| *Penicillium jensenii* |
| *Penicillium kojigenum* |
| *Penicillium lanosum* |
| *Penicillium lividum* |
| *Penicillium madriti* |
| *Penicillium mali* |
| *Penicillium manginii* |
| *Penicillium marneffei* |
| *Penicillium megasporum* |
| *Penicillium melanoconidium* |
| *Penicillium meleagrinum* |
| *Penicillium melinii* |
| *Penicillium miczynskii* |
| *Penicillium minioluteum* |
| *Penicillium montanense* |
| *Penicillium nalgiovense* |
| *Penicillium namyslowskii* |
| *Penicillium neoechinulatum* |
| *Penicillium novae-zeelandiae* |
| *Penicillium ochrochloron* |
| *Penicillium olsonii* |
| *Penicillium oxalicum* |
| *Penicillium paneum* |
| *Penicillium paraherquei* |
| *Penicillium paxilli* |
| *Penicillium phoeniceum* |
| *Penicillium piceum* |
| *Penicillium pimiteouiense* |
| *Penicillium pinophilum* |
| *Penicillium polonicum* |
| *Penicillium purpurescens* |
| *Penicillium purpurogenum* |
| *Penicillium quercetorum* |
| *Penicillium raciborskii* |
| *Penicillium radicum* |
| *Penicillium raperi* |
| *Penicillium resedanum* |
| *Penicillium restrictum* |
| *Penicillium ribeum* |
| *Penicillium rivolii* |
| *Penicillium rolfsii* |
| *Penicillium roqueforti* |
| *Penicillium roseopurpureum* |
| *Penicillium rugulosum* |
| *Penicillium sartoryi* |
| *Penicillium scabrosum* |
| *Penicillium sclerotigenum* |
| *Penicillium simplicissimum* |
| *Penicillium skrjabinii* |
| *Penicillium solitum* |
| *Penicillium soppii* |
| *Penicillium spinulosum* |
| *Penicillium steckii* |
| *Penicillium subarcticum* |
| *Penicillium sublateritium* |
| *Penicillium sumatrense* |
| *Penicillium swiecickii* |
| *Penicillium terrestre* |
| *Penicillium thiersii* |
| *Penicillium thomii* |
| *Penicillium toxicarium* |
| *Penicillium tricolor* |
| *Penicillium turbatum* |
| *Penicillium urticae* |
| *Penicillium waksmanii* |
| *Penicillium variabile* |
| *Penicillium velutinum* |
| *Penicillium venetum* |
| *Penicillium verruculosum* |
| *Penicillium westlingii* |
| *Penicillium vinaceum* |
| *Penicillium virgatum* |
| *Penicillium viridicatum* |
| *Penicillium vulpinum* |
| *Petromyces albertensis* |
| *Petromyces alliaceus* |
| *Petromyces muricatus* |
| *Ramichloridium mackenziei* |
| *Rhinocladiella anceps strain* |
| *Rhinocladiella aquaspersa* |
| *Rhinocladiella atrovirens* |
| *Rhinocladiella basitona* |
| *Rhinocladiella basitonum* |
| *Rhinocladiella fasciculata* |
| *Rhinocladiella mackenziei* |
| *Rhinocladiella phaeophora* |
| *Rhinocladiella similis* |
| *Sclerocleista ornata* |
| *Stachybotrys bisbyi* |
| *Stachybotrys chlorohalonata* |
| *Stachybotrys cylindrospora* |
| *Stachybotrys dichroa* |
| *Stachybotrys echinata* |
| *Stachybotrys elegans* |
| *Stachybotrys kampalensis* |
| *Stachybotrys longispora* |
| *Stachybotrys microspora* |
| *Stachybotrys nephrospora* |
| *Stachybotrys oenanthes* |
| *Stachybotrys parvispora* |
| *Thermoascus aurantiacus* |
| *Thermoascus aurantiacus* |
| *Thermoascus crustaceus* |
| *Trichoderma aggressivum* |
| *Trichoderma album* |
| *Trichoderma asperellum* |
| *Trichoderma atroviride* |
| *Trichoderma aureoviride* |
| *Trichoderma austrokoningii* |
| *Trichoderma brevicompactum* |
| *Trichoderma caribbaeum* |
| *Trichoderma cf. citrinoviride* |
| *Trichoderma cf. harzianum* |
| *Trichoderma cf. stilbohypoxyli* |
| *Trichoderma cf. stramineum* |
| *Trichoderma cf. viridescens* |
| *Trichoderma citrinoviride* |
| *Trichoderma croceum* |
| *Trichoderma dingleyeae* |
| *Trichoderma dorotheae* |
| *Trichoderma effusum* |
| *Trichoderma erinaceum* |
| *Trichoderma fasciculatum* |
| *Trichoderma fertile* |
| *Trichoderma ghanense* |
| *Trichoderma hamatum* |
| *Trichoderma helicum* |
| *Trichoderma inhamatum* |
| *Trichoderma intricatum* |
| *Trichoderma konilangbra* |
| *Trichoderma koningiopsis* |
| *Trichoderma longibrachiatum* |
| *Trichoderma longipile* |
| *Trichoderma oblongisporum* |
| *Trichoderma ovalisporum* |
| *Trichoderma parceramosum* |
| *Trichoderma petersenii* |
| *Trichoderma pubescens* |
| *Trichoderma rogersonii* |
| *Trichoderma rossicum* |
| *Trichoderma saturnisporum* |
| *Trichoderma sinensis* |
| *Trichoderma spirale* |
| *Trichoderma stilboxypoxyli* |
| *Trichoderma strigosum* |
| *Trichoderma stromaticum* |
| *Trichoderma taiwanense* |
| *Trichoderma taxi* |
| *Trichoderma tomentosum* |
| *Trichoderma velutinum* |
| *Trichoderma viride* |
|  |

**Species detected by the *Asp/Pen* DNA test**

| *Aspergillus bridgeri* |
| --- |
| *Aspergillus caelatus* |
| *Aspergillus cf. Fumigatus* |
| *Aspergillus cf. tamarii* |
| *Aspergillus conicus* |
| *Aspergillus crystallinus* |
| *Aspergillus elegans* |
| *Aspergillus ellipticus* |
| *Aspergillus flavofurcatus* |
| *Aspergillus flavus* |
| *Aspergillus fumigatus* |
| *Aspergillus gracilis* |
| *Aspergillus heteromorphus* |
| *Aspergillus kambarensis* |
| *Aspergillus lanosus* |
| *Aspergillus malodoratus* |
| *Aspergillus melleus* |
| *Aspergillus niger* |
| *Aspergillus ochraceus* |
| *Aspergillus oryzae* |
| *Aspergillus ostianus* |
| *Aspergillus paradoxus* |
| *Aspergillus parasiticus* |
| *Aspergillus penicillioides* |
| *Aspergillus petrakii* |
| *Aspergillus phialisepticus* |
| *Aspergillus phoenicis* |
| *Aspergillus proliferans* |
| *Aspergillus pseudotamarii* |
| *Aspergillus restrictus* |
| *Aspergillus sclerotiorum* |
| *Aspergillus sojae* |
| *Aspergillus subolivaceus* |
| *Aspergillus sulphureus* |
| *Aspergillus tamarii* |
| *Aspergillus terricola* |
| *Aspergillus thomii* |
| *Davidiella tassiana* |
| *Eupenicillium baarnense* |
| *Eupenicillium bovifimosum* |
| *Eupenicillium brefeldianum* |
| *Eupenicillium cinnamopurpureum* |
| *Eupenicillium crustaceum* |
| *Eupenicillium egyptiacum* |
| *Eupenicillium ehrlichii* |
| *Eupenicillium javanicum* |
| *Eupenicillium lapidosum* |
| *Eupenicillium levitum* |
| *Eupenicillium limosum* |
| *Eupenicillium reticulisporum* |
| *Eupenicillium stolkiae* |
| *Eupenicillium terrenum* |
| *Eurotium amstelodami* |
| *Eurotium athecium* |
| *Eurotium carnoyi* |
| *Eurotium chevalieri* |
| *Eurotium cristatum* |
| *Eurotium echinulatum* |
| *Eurotium herbariorum* |
| *Eurotium intermedium* |
| *Eurotium leucocarpum* |
| *Eurotium medium* |
| *Eurotium niveoglaucum* |
| *Eurotium pseudoglaucum* |
| *Eurotium repens* |
| *Eurotium rubrum* |
| *Eurotium tonophilum* |
| *Hemicarpenteles paradoxus* |
| *Neosartorya aureola* |
| *Neosartorya fennelliae* |
| *Neosartorya fischeri* |
| *Neosartorya glabra* |
| *Neosartorya quadricincta* |
| *Neosartorya spathulata* |
| *Neosartorya spinosa* |
| *Paecilomyces variotii* |
| *Paracoccidioides cerebriformis* |
| *Penicillium aethiopicum* |
| *Penicillium atramentosum* |
| *Penicillium atrovenetum* |
| *Penicillium aurantiogriseum* |
| *Penicillium boreae* |
| *Penicillium brevicompactum* |
| *Penicillium camemberti* |
| *Penicillium canariense* |
| *Penicillium canescens* |
| *Penicillium capsulatum* |
| *Penicillium carneum* |
| *Penicillium chermesinum* |
| *Penicillium chrysogenum* |
| *Penicillium cinerascens* |
| *Penicillium citreonigrum* |
| *Penicillium clavigerum* |
| *Penicillium commune* |
| *Penicillium concentricum* |
| *Penicillium coprobium* |
| *Penicillium coprophilum* |
| *Penicillium cordubense* |
| *Penicillium corylophilum* |
| *Penicillium crustosum* |
| *Penicillium cyaneum* |
| *Penicillium daleae* |
| *Penicillium digitatum* |
| *Penicillium dipodomyicola* |
| *Penicillium donkii* |
| *Penicillium echinulatum* |
| *Penicillium expansum* |
| *Penicillium farinosum* |
| *Penicillium freii* |
| *Penicillium fuscum* |
| *Penicillium gladioli* |
| *Penicillium glandicola* |
| *Penicillium griseofulvum* |
| *Penicillium hirsutum* |
| *Penicillium implicatum* |
| *Penicillium isariiforme* |
| *Penicillium janthinellum* |
| *Penicillium jensenii* |
| *Penicillium madriti* |
| *Penicillium mali* |
| *Penicillium melinii* |
| *Penicillium namyslowskii* |
| *Penicillium olsonii* |
| *Penicillium oxalicum* |
| *Penicillium paneum* |
| *Penicillium polonicum* |
| *Penicillium raperi* |
| *Penicillium sabulosum* |
| *Penicillium sclerotigenum* |
| *Penicillium spinulosum* |
| *Penicillium subarcticum* |
| *Penicillium thomii* |
| *Penicillium turbatum* |
| *Penicillium velutinum* |
| *Penicillium westlingii* |
| *Penicillium viridicatum* |
| *Penicillium vulpinum* |
| *Petromyces albertensis* |
| *Petromyces alliaceus* |
| *Thermoascus aurantiacus* |

**Species detected by the *Streptomyces* DNA test**

| *Micromonospora megalomicea* |
| --- |
| *Streptomyces achromogenes subsp. rubradiris* |
| *Streptomyces acidoresistans* |
| *Streptomyces acrimycini* |
| *Streptomyces ahygroscopicus subsp. wuzhouensis* |
| *Streptomyces akiyoshiensis* |
| *Streptomyces albidoflavus* |
| *Streptomyces albidus* |
| *Streptomyces albolongus* |
| *Streptomyces alboniger* |
| *Streptomyces albovinaceus* |
| *Streptomyces alboviridis* |
| *Streptomyces albus subsp. albus* |
| *Streptomyces almquistii* |
| *Streptomyces althioticus* |
| *Streptomyces anthocyanicus* |
| *Streptomyces antibioticus* |
| *Streptomyces anulatus* |
| *Streptomyces argenteolus* |
| *Streptomyces atratus* |
| *Streptomyces atroolivaceus* |
| *Streptomyces aurantiacus* |
| *Streptomyces aurantiogriseus* |
| *Streptomyces aureocirculatus* |
| *Streptomyces aureofaciens* |
| *Streptomyces aureoverticillatus* |
| *Streptomyces aureus* |
| *Streptomyces baarnensis* |
| *Streptomyces bacillaris* |
| *Streptomyces badius* |
| *Streptomyces bikiniensis* |
| *Streptomyces bobili* |
| *Streptomyces bottropensis* |
| *Streptomyces cacaoi subsp. cacaoi* |
| *Streptomyces caeruleus* |
| *Streptomyces caesius* |
| *Streptomyces californicus* |
| *Streptomyces candidus* |
| *Streptomyces caniferus* |
| *Streptomyces canus* |
| *Streptomyces caviscabies* |
| *Streptomyces cavourensis subsp. cavourensis* |
| *Streptomyces cavourensis subsp. washingtonensis* |
| *Streptomyces celluloflavus* |
| *Streptomyces cf. Griseus* |
| *Streptomyces cinereorectus* |
| *Streptomyces cinereoruber* |
| *Streptomyces cinereoruber subsp. Cinereoruber* |
| *Streptomyces cinereus* |
| *Streptomyces cinnamocastaneus* |
| *Streptomyces ciscaucasicus* |
| *Streptomyces clavifer* |
| *Streptomyces coelescens* |
| *Streptomyces coelicoflavus* |
| *Streptomyces coelicolo* |
| *Streptomyces collinus* |
| *Streptomyces cremeus* |
| *Streptomyces cyaneofuscatus* |
| *Streptomyces cyaneus* |
| *Streptomyces cyanocolor* |
| *Streptomyces cyanogenus* |
| *Streptomyces danangensis* |
| *Streptomyces diastaticus subsp. Ardesiacus* |
| *Streptomyces diastaticus subsp. Diastaticus* |
| *Streptomyces drozdowiczii* |
| *Streptomyces ederensis* |
| *Streptomyces erumpens* |
| *Streptomyces erythrogriseus* |
| *Streptomyces exfoliatus* |
| *Streptomyces fimicarius* |
| *Streptomyces finlayi* |
| *Streptomyces flaveus* |
| *Streptomyces flavidofuscus* |
| *Streptomyces flavogriseus* |
| *Streptomyces flavolimosus* |
| *Streptomyces flavovirens* |
| *Streptomyces floridae* |
| *Streptomyces fradiae* |
| *Streptomyces fragilis* |
| *Streptomyces fulvorobeus* |
| *Streptomyces galilaeus* |
| *Streptomyces gelaticus* |
| *Streptomyces globisporus subsp. Globisporus* |
| *Streptomyces globosus* |
| *Streptomyces glomeroaurantiacus* |
| *Streptomyces goraiensis* |
| *Streptomyces gougerotii* |
| *Streptomyces graminearus* |
| *Streptomyces graminofaciens* |
| *Streptomyces griseinus* |
| *Streptomyces griseobrunneus* |
| *Streptomyces griseoincarnatus* |
| *Streptomyces griseolus* |
| *Streptomyces griseoplanus* |
| *Streptomyces griseorubens* |
| *Streptomyces griseus* |
| *Streptomyces griseus subsp. griseus* |
| *Streptomyces griseus subsp. rhodochrous* |
| *Streptomyces halstedii* |
| *Streptomyces heliomycini* |
| *Streptomyces hirsutus* |
| *Streptomyces humidus* |
| *Streptomyces humiferus* |
| *Streptomyces intermedius* |
| *Streptomyces kanamyceticus* |
| *Streptomyces kitasatoensis* |
| *Streptomyces kurssanovii* |
| *Streptomyces labedae* |
| *Streptomyces laceyi* |
| *Streptomyces lateritius* |
| *Streptomyces lavendulae subsp. Lavendulae* |
| *Streptomyces lazureus* |
| *Streptomyces lienomycini* |
| *Streptomyces lividans* |
| *Streptomyces lohii* |
| *Streptomyces longisporoflavus* |
| *Streptomyces longispororuber* |
| *Streptomyces luteosporeus* |
| *Streptomyces maritimus* |
| *Streptomyces marokkonensis* |
| *Streptomyces matensis* |
| *Streptomyces mediolani* |
| *Streptomyces microflavus* |
| *Streptomyces moderatus* |
| *Streptomyces mutomycini* |
| *Streptomyces naraensis* |
| *Streptomyces narbonensis* |
| *Streptomyces nodosus subsp. asukaensis* |
| *Streptomyces novaecaesareae* |
| *Streptomyces olivaceus* |
| *Streptomyces olivochromogenes* |
| *Streptomyces olivoviridis* |
| *Streptomyces ornatus* |
| *Streptomyces ostreogriseus* |
| *Streptomyces paresii* |
| *Streptomyces parvus* |
| *Streptomyces peucetius* |
| *Streptomyces peucetius subsp. caesius* |
| *Streptomyces phaeochromogenes* |
| *Streptomyces phaeofaciens* |
| *Streptomyces piomogenus* |
| *Streptomyces platensis subsp. robigocidicus* |
| *Streptomyces pluricolorescens* |
| *Streptomyces praecox* |
| *Streptomyces pseudogriseolus* |
| *Streptomyces pseudogriseolus subsp. glucofermentans* |
| *Streptomyces pseudovenezuelae* |
| *Streptomyces pulveraceus* |
| *Streptomyces puniceus* |
| *Streptomyces resistomycificus* |
| *Streptomyces rimosus subsp. rimosus* |
| *Streptomyces rochei i* |
| *Streptomyces rosealbus* |
| *Streptomyces roseochromogenus* |
| *Streptomyces roseoflavus* |
| *Streptomyces rubiginosohelvolus* |
| *Streptomyces rubrogriseus* |
| *Streptomyces rutgersensis* |
| *Streptomyces rutgersensis subsp. rutgersensis* |
| *Streptomyces sanglieri* |
| *Streptomyces scabiei* |
| *Streptomyces seoulensis* |
| *Streptomyces setonii* |
| *Streptomyces sindenensis* |
| *Streptomyces spiroverticillatus* |
| *Streptomyces stelliscabiei* |
| *Streptomyces tanashiensis* |
| *Streptomyces tendae* |
| *Streptomyces termitum* |
| *Streptomyces thermophilus* |
| *Streptomyces tricolor* |
| *Streptomyces tritolerans* |
| *Streptomyces tsusimaensis* |
| *Streptomyces turgidiscabies* |
| *Streptomyces umbrinus* |
| *Streptomyces variabilis* |
| *Streptomyces vastus* |
| *Streptomyces vietnamensis* |
| *Streptomyces vinaceus* |
| *Streptomyces violaceolatus* |
| *Streptomyces violaceoruber* |
| *Streptomyces violaceorubidus* |
| *Streptomyces viridochromogenes* |
| *Streptomyces xantholiticus* |
| *Streptomyces xylophagus* |
| *Streptomyces yanii* |
| *Streptomyces yerevanensis* |
